# Mechanistic insight into the formation of colloidal WS_2_ nanoflakes in hot alkylamine media†

**DOI:** 10.1039/c9na00279k

**Published:** 2019-05-24

**Authors:** Riccardo Scarfiello, Andrea Cesari, Davide Altamura, Sofia Masi, Concetta Nobile, Federica Balzano, Cinzia Giannini, Vincenzo Grillo, Amir H. Tavabi, Rafal E. Dunin-Borkowski, Gloria Uccello-Barretta, P. Davide Cozzoli, Aurora Rizzo

**Affiliations:** CNR NANOTEC, Institute of Nanotechnology c/o Campus Ecotecne, via Monteroni 73100 Lecce Italy riccardo.scarfiello@nanotec.cnr.it aurora.rizzo@nanotec.cnr.it; Department of Chemistry and Industrial Chemistry, University of Pisa via Moruzzi 13 56124 Pisa Italy; IC CNR, Institute of Crystallography via Amendola 122/O I-70126 Bari Italy; Centro S3, CNR Istituto Nanoscienze via Campi 213/A 41125 Modena Italy; Ernst Ruska-Centre for Microscopy and Spectroscopy with Electrons, Forschungszentrum Jülich 52425 Julich Germany; Department of Mathematics and Physics E. De Giorgi, University of Salento via per Arnesano Lecce 73100 Italy; UdR INSTM di Lecce, Università del Salento c/o, Campus Ecotekne, via Arnesano 73100 Lecce Italy

## Abstract

Developing convenient and reliable synthetic methodologies for solution processable 2D layered ultrathin nanostructures with lateral size control is one of the major challenges for practical applications. In this study, a rational understanding a long-chain amphiphilic surfactant assisted non-hydrolytic synthesis that is able to generate dimension-controllable 2D-WS_2_ nanocrystal flakes in a single-step protocol is proposed. The evolution of the starting soft organic–inorganic lamellar template into ultrathin few-layer 2D-WS_2_ nanostructures with lateral size modulation over a range between 3 and 30 nm is monitored. The initial formation of WS_2_ nanoseeds occurs in a self-assembled sacrificial precursor source, acting as a template, where larger two-dimensional nanostructures can grow without undergoing significant thickness variation. Overall, the chemical nature and steric hindrance of the alkylamines are essential to modulate the reactivity of such WS_2_ nanoclusters, which correlate with the lateral size of the resulting nanoflakes.

## Abstract 2

Non-hydrolytic synthesis assisted by long-chain amphiphilic surfactant is exploited to generate dimension-controllable 2D-WS_2_ nanoflakes in a single-step protocol, where the chemical nature and steric hindrance of the alkylamine are the key points to modulate the lateral size finally achieved.

## Graphical abstract

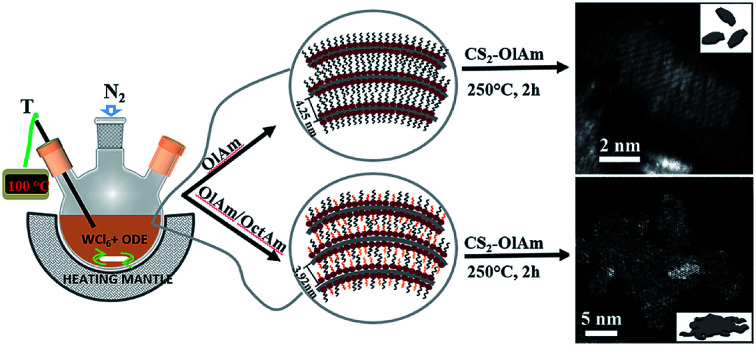

## Introduction

Among the variety of materials with layered structure, transition metal dichalcogenides (TMDs) have attracted exceptional interest thanks to their unique thickness and structure-dependent physicochemical properties.^1–4^ The emergence of surfactant assisted non-hydrolytic synthesis has recently paved the way for the production of solution processable freestanding TMD colloidal nanocrystals in a thickness-controlled regime with good crystallinity, monodispersity, phase purity or possibly hetero-phase structures, and controlled edge functionalization.^5^ At this early stage, to push further the synthesis of ultrathin 2D layered TMD nanocrystals with desired physicochemical properties, developing more effective synthetic routes along with close control of formation mechanisms is imperative.^6,7^ In general, solvothermal synthetic methods proposed so far have allowed the synthesis of colloidal WS_2_ monolayers produced in the less common distorted octahedral structure (1T′-WS_2_) or in their more aggregated semiconducting nanostructures (2H-WS_2_).^8^ Herein we report an alternative mechanism for the synthesis of solution processable 2D-WS_2_ nanoflakes (NFLs) with lateral size modulation over a range between 3 and 30 nm, without inducing significant modifications in the crystal structure. We explore the ability of long-chain amphiphilic amines to direct the growth of a layered hybrid tungsten-based intermediate, in which tungsten sulfide small nuclei can organize to produce NFLs. The investigated synthetic approach is of great general interest, since liquids containing diphilic molecules (hydrophilic terminal atomic groups bonded to lipophilic hydrocarbon chains) can structurally organize into a double layer efficiently serving as a soft template for hydrophilic compounds, such as inorganic ions, which can arrange interacting with each other, and form “flat” crystals chemically guided by the interlayer space.^9,10^ This mechanism is often connected to another spontaneous kinetically driven process, namely the so-called “oriented attachment” that takes place when 0D or 2D building blocks connect at specific crystallographic surfaces, reducing the overall energy of the system by removing the surface energy associated with unsaturated bonds.^11–13^ The role of diphilic molecules in this case is essential, since it might not only influence the stability or modulate the reactivity of metal complexes present in solution, but also selectively cover certain crystal facets, thus preventing aggregation of these facets. Similar synthetic schemes have been exploited for the formation of cadmium and lead chalcogenide nanocrystals in long-chain primary alkylamines in the *in situ* generation of lamellar mesophase intermediates^11,14–17^ or the gradual crystallization of inorganic nanocrystal building blocks present in the 2D soft template.^18^

A long-chain amphiphilic amine has been used as well as a template to direct the self-assembly of rhenium selenide sub-nm clusters into a layered hybrid material by linking to the relative [Re_*x*_Se_*y*_Cl_*z*_] building blocks.^19^

Herein, we rationalize the chemical pathway underlying the tunability of NFL lateral dimensions through a comprehensive study of morphological, structural, optical and chemical evolution of the precursors into 2D-WS_2_ NFLs under relatively mild reaction conditions (250 °C) and in different amine environments. We select tungsten disulfide as a case study due to its more extended applicability in numerous fields.^20,21^ Monitoring the reaction progress using X-ray diffraction (XRD), transmission electron microscopy (TEM), nuclear magnetic resonance (NMR), UV-vis and Fourier Transform Infrared (FT-IR) spectroscopy, we find that the initially formed tungsten sulfide nano-nuclei, which act as building blocks for the WS_2_-NFL evolution, are stabilized by amine and additional chloride ligands. The hydrophobic tail–tail interaction of amphiphiles assists the self-assembly of such tungsten sulfide nuclei, which eventually evolve into 2D-WS_2_ ultrathin NFLs with different lateral dimensions. The spontaneous organization of long-chain aliphatic amines coordinating with the inorganic precursor indeed generates a soft mesophase-template, which acts as a pseudoplanar reaction gallery, where the *in situ* WS_2_ nuclei form and then further evolve into extended NFL structures. Importantly, both the stability of these building blocks and the kinetics involved during the nanocrystal generation are connected with the nature and steric hindrance of ligands, reactivity of the metal precursor, temperature and concentration, which become the key elements to understand and eventually predict the 2D-WS_2_ NFL size and morphology finally achieved. Our study presents a guideline for the selection of surfactants and precursors in order to promote the formation of ultrathin TMD nanocrystals with controlled lateral dimensions, providing an insight into the rationalization of chemical mechanisms involved in the formation of colloidal TMDs.

## Results and discussion

**Fig. 1:**
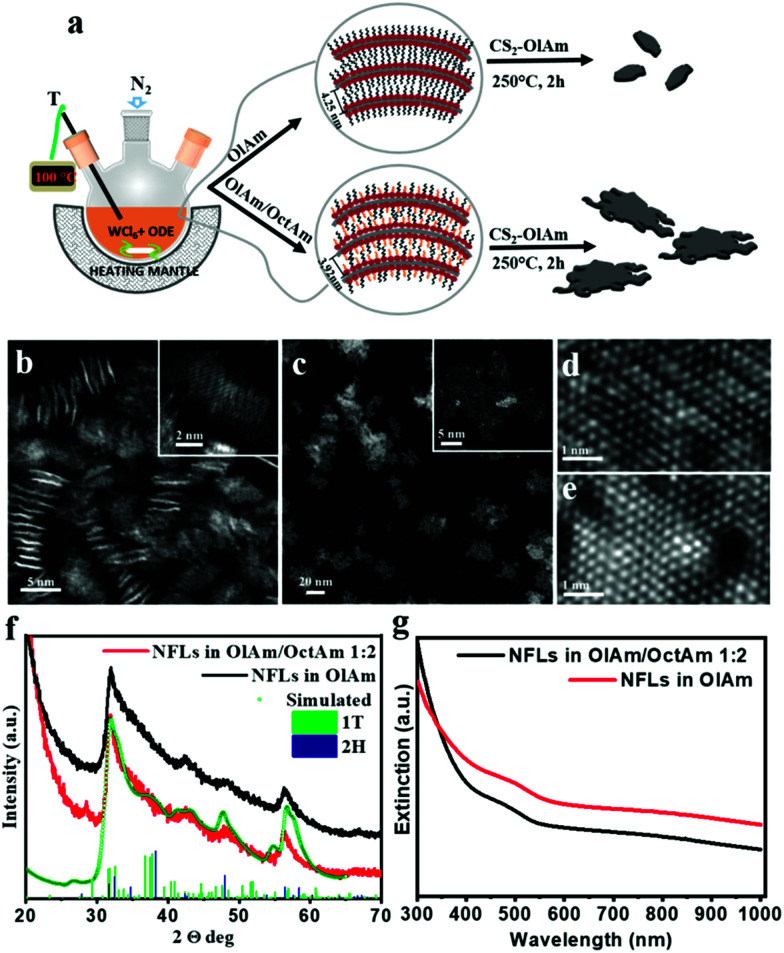
(a) Schematic illustration of the surfactant assisted wet-chemical approach for ultrathin mono-/few-layer 2D-WS_2_ NFLs in two different amine contents. (b) Low magnification HAADF STEM overviews of ∼3–5 nm and (c) ∼30 nm NFLs, and their higher magnification insets representing typical individual NFLs in the two sample populations, respectively. Atomic-resolution HAADF STEM images of NFL regions arranged in 1T′ (d) and 2H crystal symmetry (e) down the <100> and <0001> axis respectively. (f) Comparison between experimental and simulated XRD patterns based on the Debye scattering equation; (g) UV-vis absorption spectra of 2D-WS_2_ NFL CHCl_3_ solutions obtained in sole OlAm (red) and and OlAm/OctAm mixture at a 1 : 2 molar ratio (black).

In a typical synthesis, alkyl amines (oleylamine or OlAm, octylamine or OctAm) were used as high-boiling point coordinating solvents. The synthetic strategy is sketched in Fig. 1a, summarizing how the reaction conditions can be varied to access 2D-WS_2_ NFLs with the same crystalline features, but consistently different lateral dimensions. Briefly, the tungsten precursor (WCl_6_) was first dispersed in degassed octadecene (ODE) and then dissolved in the appropriate amine or amine mixture (see the Methods section), which resulted in a complex of tungsten(vi) chloride coordinated by the amine (W-amine) (Scheme S1†), as indicated by the yellowish color of the corresponding solution.^22^ We have selected CS_2_ as a sulfur source, since no radical species are formed when CS_2_ in oleylamine is heated as instead occurs for other sulphur precursors, such as elemental S in OlAm. The generation of radical species indeed promotes the degradation of the structural integrity of layered nanocrystals.^23^

We have found that during our synthetic screening by using a single surfactant (OlAm), lateral size tuning could not be achieved by varying the surfactant to precursor ratio or precursor concentration in solution. We attributed this experimental observation to the high reactivity of CS_2_. Thus, we have experimented an alternative way to guide the reaction towards morphology tuning, which consisted of the use of a co-surfactant, octylamine (OctAm). OctAm is indeed prone to form lamellar complexes with metal halides.^10^ Thus, OctAm can be exploited, in combination with OlAm, for the solvothermal synthesis of bi-dimensional nanostructures, to promote the lateral growth and eventually obtain mono- to few-layer nanocrystal structures by taking advantage of the reduced effective contact and enhanced steric repulsion between lamellas. In addition, OctAm has been recently identified to increase the reactivity of the metal precursor,^24^ increasing its conversion rate, which can influence the morphology of the final product.

The amine composition was found to be the key prerequisite to induce significant morphological modification without inducing any structural variation (*vide infra*).^25^ Carbon disulfide (CS_2_) was identified as a suitable sulfur precursor source that, in the presence of primary aliphatic amines, effectively reacts with metal chloride complexes already under mild reaction conditions.^23,26^ Because of its high volatility and reactivity, CS_2_ was dispersed in an excess of OlAm before being injected into a WCl_6_/amines/ODE mixture. With this strategy we exploited the reactivity between CS_2_ and a primary amine, assuming that dithiocarbamate acid (not isolated intermediate)^26^ is readily formed by the nucleophilic attack of a primary amine to CS_2_ already at room temperature during the CS_2_–OlAm solution preparation. The excess of oleylamine present in the reaction mixture and the thermal energy supplied to the system (130 °C) after the injection would induce the rapid addition of a second molecule of amine, leading to an *in situ* release of H_2_S (Scheme S2†). The solution was aged at 130 °C for one more hour, and then slowly heated up to 250 °C, leading to a gradual color change to black suggesting the nucleation and growth of 2D-WS_2_ NFLs.^8^ The reaction progress is based on the formation of ultrasmall nuclei that, directed by the alkyl amine environment, have the tendency to attach to each other in order to minimize the overall surface energy of the system. Considering this, in our synthetic methodology, we adopted the lowest synthetic temperature for 2D WS_2_ NFLs, when compared with other protocols described in the literature.^8,27^ This experimental choice was aimed at the suppression of the “out-of-plane” growth to favor the “in-plane growth in order to achieve single-to-bi layer thick colloidal nanostructures, driving the reaction in a kinetic regime. In other words, when we performed the reaction at a higher temperature than 250 °C, only colloidally unstable multilayered structures were produced. From this, we deduced that the ultrasmall nuclei stability was compromised and a controlled building block attachment was suppressed, leading to a gradual transition of the growth mode from a stepwise growth regime (*i.e.*, based on the addition of preformed small nuclei to the growing nanocrystals) to a continuous growth regime; the latter would allow the generation of unstable multilayered structures.

High-angle annular dark-field (HAADF) scanning transmission electron microscopy (STEM) images, through the Z-contrast mechanism, enabled direct observation of the W and S atom columns and their arrangement in the crystal lattice, its layered structure and the overall nanocrystal morphology. Low magnification HAAF-STEM images in Fig. 1b and c give overviews of the as-synthesized nanostructures achieved in the presence of the sole OlAm and a mixture of OlAm/OctAm, respectively. It could be readily observed that individual ultrathin NFLs with very different lateral sizes could be obtained, with average edge dimensions of 3–5 nm for the sole OlAm and up to 30 nm for the OlAm/OctAm mixture. both types of WS_2_ NFLs were characterized by irregular edges and a layered structure, with a basal layer much more extended with respect to the top layers (see the inset of Fig. 1b and c). The presence of many NFLs standing transversally on the carbon support grid (Fig. 1b) clearly showed their mono-/few-layer identity. Atomic-resolution HAADF STEM images (Fig. 1d and e) highlight the crystal phases recognizable in all the NFL samples: the metallic distorted 1T′ (isostructural to rhenium disulphide 1T structure, ICSD code: 75459)^12,13^ and the semiconducting 2H hexagonal crystal phase (ICSD code: 202366).^28^ Experimental XRD patterns, reported in Fig. 1f, were characterized by broad convoluted peaks between 20° < 2*θ* < 70°, consistent with the nanosized dimensions of the crystals, and confirmed the coexistence of 1T′ and 2H in the heterophase nano-structures for both samples. The complete kinetic and thermodynamic model for the formation of colloidal WS_2_ NFL polytypic heterostructures in which vertically and laterally interconnected 1H/2H and metallic 1T′ domains coexist will be exhaustively reported elsewhere. A rough estimation of the NFL sample phase composition was attempted by comparing the experimental data against a linear combination of the scattering profiles relevant to NFLs with different sizes and crystalline phases, 1T′ or 2H. All scattering profiles were calculated based on the Debye equation^29,30^ and normalized to the unit cell volume so that they were on the same scale of intensity. By changing the relative abundance of the different components in the calculation, it was found that the best agreement could be obtained for the presence on average of ∼86% of very small 1T′ and ∼14% of larger 2H domains, both being 2D crystalline, *i.e.* with one or at most two unit cells along one of the three main crystallographic axes (*a* and *c* for 1T′ and 2H respectively), and about 10 or 40 unit cells in the lateral dimension, respectively. Details of the flowchart of pattern simulation are reported in the ESI (Fig. S1–S5†). The optical extinction spectra in solutions of the corresponding samples, Fig. 1g, displayed a featureless profile, as expected for hetero-phase NFLs, matching semiconducting 2H and metallic 1T′ motifs.^8^

**Fig. 2:**
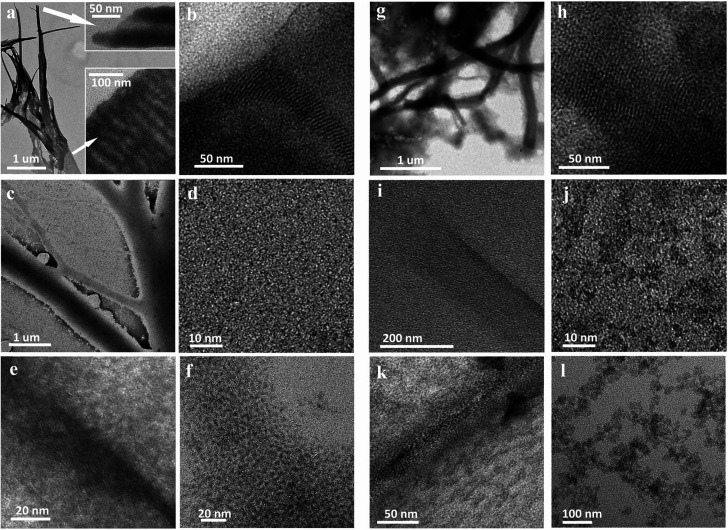
Representative TEM images showing the main steps of the morphological evolution of the reaction products isolated during the synthesis of WS_2_-NFLs obtained in sole OlAm (a–f) and NFLs obtained in OlAm/OctAm mixture with 1 : 2 molar ration (g–l). (a and b) Representative images of WCl_6_/ODE/OlAm (0.25 : 3 : 3 mmol) obtained by the precipitation of aliquots withdrawn after heating at 130 °C for 1 h under vacuum (20 mTor); (c and d) Images obtained on the product precipitated from reaction aliquots withdrawn after injection of the CS_2_–OlAm solution and heating up to 215 °C; (e and f) obtained on the products precipitated from reaction aliquots withdrawn as a soon as the system reaches 250 °C. (g and h) Representative TEM images of WCl_6_/ODE/OlAm/OctAm (0.25 : 3 : 3 : 6) obtained on the products precipitated from reaction aliquots withdrawn after degassing for 1 h under vacuum (20 mTor) at room temperature; (i and j) obtained on the product precipitated from reaction aliquots withdrawn after injection of the CS_2_–OlAm solution and heating up to 215 °C; (k and l) obtained on the products precipitated from reaction aliquots withdrawn as soon as the system reaches 250 °C.

To shine light on the formation mechanism of such ultrathin nanostructures from their origin, we have monitored the morphological, chemical, structural and optical evolution of the two different synthesis products at different stages of the reaction course. Low magnification TEM inspection showed micrometer scale extended hybrid amine/tungstate(vi) chloride lamellas (Fig. 2a and g), which were spontaneously formed at the beginning of the reaction when primary amines were introduced into the WCl_6_–ODE dispersion, corroborating the hypothesis depicted in Fig. 1a.^14^ The presence of organic/inorganic lamellas was also supported by small-angle XRD reported in Fig. S6.†

We found that by treating WCl_6_ in ODE with the sole OlAm, the resulting lamellar hybrid mesophase could be easily isolated and characterized. Areas with wrinkles and bends were clearly visible, suggesting the formation of open-ended multilayered cylindrical structures (Fig. 2a and b). We noticed that treating WCl_6_ in ODE with OlAm/OctAm (Fig. 2g and h) also resulted in a lamellar structure, but apparently less resistant toward precipitation/dissolution cycles and electron beam exposure during TEM investigation, which could be still identified, albeit with a less definable morphology. This soft mesophase-template was formed by spontaneous organization of long-chain aliphatic amines coordinating with the inorganic precursor, resulting in inorganic–organic complexes, which hierarchically self-assembled into planar or multiwalled tubular sacrificial mesostructures.^12,31^ The templates acted as a pseudoplanar reaction gallery, where the *in situ* WS_2_ nuclei were readily formed, possibly embedded in it, and could further evolve into more extended NFLs.^11–13^

Upon the injection of the chalcogen precursor, CS_2_ in OlAm, the system was slowly heated up to 250 °C. In Fig. 2c, d, i and j representative micrographs, recorded from aliquots isolated while the reaction systems were heated above 215 °C, show ultrathin damaged lamellas along with small nanostructures. We hypothesize that, at this stage, the hybrid mesostructures underwent thermal decomposition into thinner single or few lamellae layers (as confirmed by the broadening of the corresponding small-angle XRD pattern, Fig. S6c and d†), in which the nucleation of WS_2_ nano-building blocks apparently took place. This hypothesis is supported by the absorption spectra (reported in Fig. S7c and d†) evidencing the presence of a peak below 400 nm, typical of ultrasmall structures, which gradually broadens and then flattens with the prolonging of reaction time.^32,33^

As soon as the systems reached the final annealing temperature of 250 °C, 2D-WS_2_ NFLs were completely formed and released into solution. Nanocrystal release occurred close to the hybrid lamellar precursors, as often captured by TEM imaging (Fig. 2e, f and j, l for OlAm and OlAm/OctAm systems, respectively). The morphology evolution suggested that the reaction carried out in an OlAm/OctAm mixture resulted in the decomposition of the lamellas, when compared to the one in sole OlAm, since the nanocrystal release took place already at 215 °C (Fig. 2j).

To elucidate the reaction pathways occurring in the synthesis of 2D-WS_2_ NFLs, and eventually connecting the chemical evolution with morphological features, we measured specific reaction steps relative to selected aliquots taken at different times with NMR spectroscopy. We mimicked the preparation of nanoparticles by using the stoichiometric amount (two equivalents) of OlAm, OctAm or the mixture of OlAm/OctAm with respect to that of CS_2_ (see Experimental for details) in order to avoid doubtful interpretations connected to the excess of surfactants (amines) and non-coordinating solvents (ODE). Moreover, since no peculiar differences have been observed in terms of reaction products when CS_2_ was reacted with OctAm and OlAm/OctAm with respect to OlAm, we report the experimental results for those two cases in the ESI (Fig. S8†) and report here the discussion about the system with sole OlAm.

**Fig. 3:**
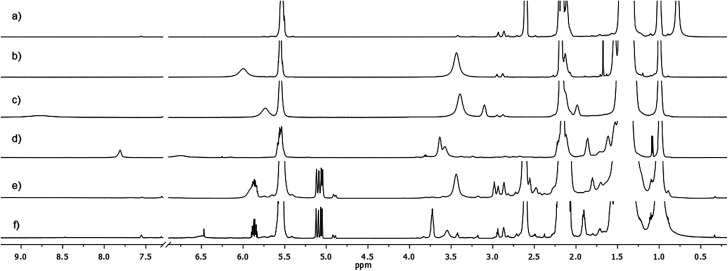
(a–f) ^1^H NMR spectrum (600 MHz, toluene-d8, 25 °C) of: (a) pure OLAM; (b) CS_2_/OlAm 1 : 2 (130 °C, 1 h); (c) WCl_6_/CS_2_/OlAm 0.25 : 4:8 (130 °C, 1 h); (d) WCl_6_/CS_2_/OlAm 0.25 : 4 : 8 (250 °C, 1 h); (e) WCl_6_/ODE/OlAm/CS_2_ under 3–5 nm WS_2_ NFL synthetic conditions (0.25 : 3 : 3 : 1; 130 °C, 1 h); (f) WCl_6_/ODE/OlAm/CS_2_ under 3–5 nm WS_2_ NFL synthetic conditions (0.25 : 3 : 3 : 1; 250 °C, 2 h).

Starting from the analysis of the product coming from the reaction between CS_2_ and two equivalents of OlAm, we clearly observed that CS_2_ was completely consumed by the amine as soon as the CS_2_/OlAm solution was injected into the reaction mixture and aged at 130 °C for 1 hour, and thus converted into a single product, *N*,*N*′-dioleylthiourea (OlAm_2_–TU), hereafter referred to as compound 1. Compound 1, characterized by its ^1^H NMR signals, when compared with the spectra of pure OlAm (Fig. 3a), produced two new broad resonances between 3.2–3.7 ppm and 5.7–6.5 ppm in a 2 to 1 integration ratio (Fig. 3b).

The former peak corresponds to methylene moieties alpha to nitrogen (–CH_2_–N), while the latter corresponds to a proton not bound to carbon, and is thus attributed to a NH amide proton. This was the consequence of two successive nucleophilic attacks of two amine units to CS_2_ with subsequent formation of symmetrical thiourea species, as corroborated by the chemical shift of 182.5 ppm (Fig. S9a†) of the quaternary carbon.^34,35^ A remarkable shortening of proton longitudinal relaxation times (*T*_1_) of –CH_2_–N (0.71 s), when compared with pure OLAM (2.50 s), reflected the molecular weight increase due to amine dimerization, upon reaction with CS_2_, with the formation of thiourea derivatives.

When WCl_6_ was included in our experimental reference model (WCl_6_/CS_2_/OlAm = 0.25 : 4 : 8 molar ratio) under the same experimental conditions (stirred for 1 h at 130 °C under N_2_), the thiourea species were once again detected at 3.39 ppm and 5.67 ppm (Fig. 3c). The relaxation parameter (*T*_1_) of OlAm_2_–TU did not change in the absence and in the presence of WCl_6_ (*T*_1_ = 0.71 s) indicating that no metal complexation took place for these species. An oleylamine hydrochloride salt like compound OlAm·HCl, see Fig. S10a and b†, referred to as compound 2 was also detected as the consequence of the presence of WCl_6_, the amount of which depended on the amount of WCl_6_ used during the reaction (Fig. S10b and c†). The experimental relaxation time of –CH_2_–N protons of OlAm·HCl was shorter (0.31 s) with respect to pure OlAm·HCl salt (0.55 s), evidencing the complexation of the species to the metal sites. we hypothesized that the complexation of OlAm with WCl_6_, necessary for the dissolution of the metal precursor, which eventually induces a partial or complete substitution of halogens with the amine moiety, results in Cl^−^ ions being coordinated to the protonated oleyl ammonium derivative (Scheme S1†). Increasing the temperature of our experimental reference model led to the formation of a new product, *i.e. N*,*N*′-dioleylcarbamimidothioic acid, compound 3 or simply (OlAm)_2_–thiol, having new methylene protons at 3.59 ppm, a remarkably deshielded proton at 7.76 ppm (Fig. 3d), not bound to carbon, and a quaternary carbon at 155.9 ppm (Fig. S9b†). Therefore, we hypothesized a tautomerization process leading to the formation of a SH group (7.76 ppm) and a quaternary imine carbon (155.9 ppm).^36–39^ The above results made easier the identification of reaction products in the mixtures containing the amine excess with respect to CS_2_ and the metal, as precisely adopted for nanocrystal synthesis in this work (Fig. 3e and f) and reported more in detail in Fig. S11.† In particular, these samples showed the signals of the thiourea species (–CH_2_–N at about 3.3 ppm and NH at 5.8 ppm), which were present since the first stage of the reaction (aged at 130 °C for 1 h, see Experimental details, Fig. S11a†) or kept at the target temperature (250 °C) for very short times (up to 10 min, Fig. S11b and c†). Prolonged heating for the whole synthesis (1 or 2 h at 250 °C, Fig. S11d and e†) in the nanoparticle systems caused the formation of both the thiourea species and its tautomeric thiolic form. It is worth underlining that NMR investigation on the CS_2_/OlAm sample kept at 250 °C for 2 hours showed the presence of 1 (OlAm_2_–TU) as the only chemical compound present in this step of synthesis (Fig. S11f†).

**Scheme 1:**
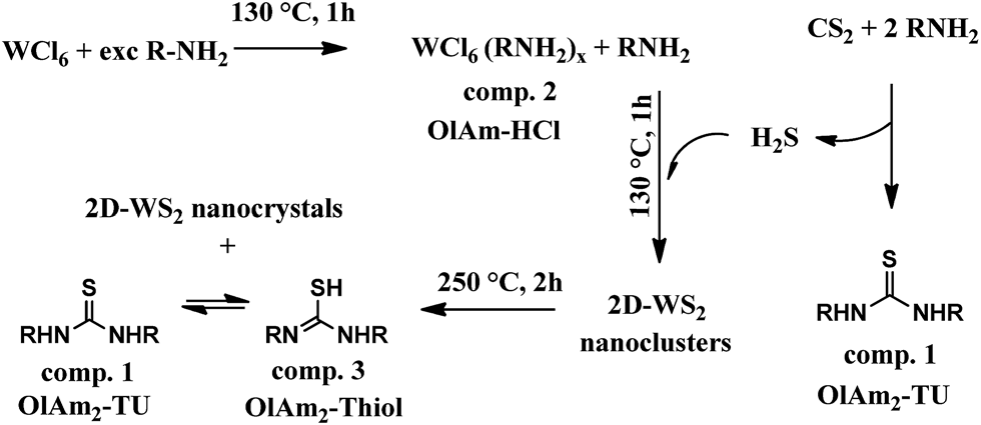
Chemical pathway proposed involved in the overall 2D-WS_2_ nanocrystal formation.

**Fig. 4:**
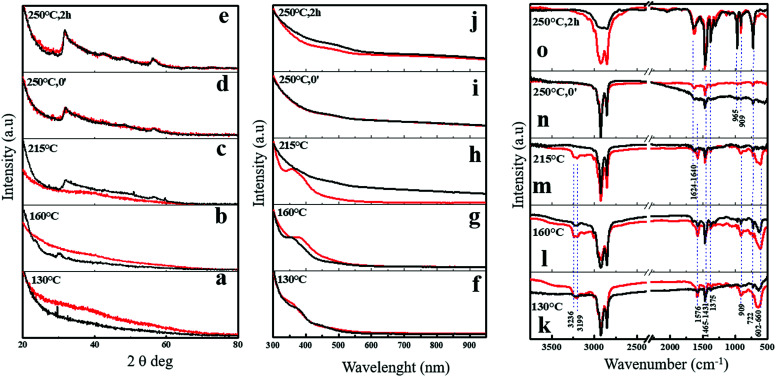
Time/temperature evolution for the main representative steps isolated during the synthesis of NFLs obtained in sole OlAm (red plots) and nanoflakes obtained in OlAm/OctAm with 1 : 2 molar ratio (red plots) and nanoflakes obtained with 1 : 2 molar ratio OlAm/OctAm (black plots). (a–e) Representative wide angle XRD profiles and (f–j) representative UV-vis extinction profiles obtained from aliquots precipitated, dried in a glove-box with nitrogen filled, and then dispersed in anhydrous CHCl_3_; representative FTIR profiles of WCl_6_/ODE/OlAm/CS_2_ (0.25 : 3 : 3 : 1) and WCl_6_/ODE/OlAm/OctAm/CS_2_ (0.25 : 3 : 3 : 6 : 1) (k–o). The red plot refers to sole OlAm synthesis and the black plot refers to 1 : 2 mmol ratio OlAm/OctAm synthesis, evidencing no substantial differences in molecular pathways underlying the heterostructured nanocrystal formation in the two different systems.

Our investigation brought us to the conclusive chemical path drawn and summarized in the sketch below (Scheme 1). In general, the reaction between an excess of primary amines with CS_2_ generated thiourea species by releasing H_2_S,^26^ which could react with WCl_6_OlAm_*x*_,^40^ already at low temperature (∼130 °C), to generate small initial nano-nuclei, detected in UV-vis spectra reported below (Fig. 4f–j, S7c and d†) and embedded in the organic/inorganic lamellar precursor. With the proceeding of the reaction those WS_2_-building blocks further evolved into the final corresponding 2D-WS_2_ NFLs. What reported in Scheme 1 can be extended to other primary aliphatic amines, for that remains valid also for OlAm/OctAm as reported in the ESI.†

To validate this hypothesis, in Scheme 1 we report the structural (XRD, Fig. 4a–e), optical (UV-vis, Fig. 4f–j) and spectroscopic (FTIR, Fig. 4k–o) evolution sequences recorded from reaction intermediates obtained from aliquots taken at scheduled time/temperature intervals, which directed the formation of both the breeds of NFLs. We have identified some representative reaction steps that are described below, with the complete measurement picture shown in the ESI.†

Wide angle XRD patterns in Fig. 4a–e, S6a and b† clearly showed the progressive growth of 2D crystalline WS_2_ flakes, with the appearance of unambiguous diffraction features starting from 250 °C or 215 °C annealing temperature for the NFL generated in the sole OlAm or in the OlAm/OctAm mixture, respectively. In this regard, we deduce that in the case of the OlAm/OctAm mixture the formation of the characteristic patterns (structural or optical) for 2D-WS_2_ is clearly anticipated when compared with the synthesis conducted in sole OlAm. What said becomes evident especially in plots recorded at 215 °C (Fig. 4c, h and m), where the black curves refer to the OlAm/OctAm mixture and already show the structural and optical profiles characteristic of the final product.

The whole process started with the previous rearrangements of nanoscale structures, as evidenced by the evolution of the small angle XRD patterns. In particular, the bottom curves in Fig. S6c and d† featured several equally spaced (in reciprocal space units) sharp peaks indicating the presence of extended multilayered structures mainly formed by inorganic tungsten layers and spaced by the organic ligands. The OctAm attenuates the rigid and bulky carbon chain of OlAm in the hybrid soft templates, resulting in the reduction of the spacing between the inorganic tungsten layers and the organic ligands^10^ that is 4.25 nm in the case of sole OlAm and 3.92 nm in OlAm/OctAm (Fig. S6c and d†). The injection of the chalcogen precursor (CS_2_) readily caused peak broadening and shifting, indicating a structural rearrangement leading to smaller (few layers) structures with smaller stacking periodicity (on average).

A complex evolution occurred during annealing in the range 160 °C–250 °C, and multilayers were still present with different though well-defined periodicities (equally spaced diffraction peaks), depending on the OctAm presence in the mixture. At 215 °C (when wide angle XRD indicates the onset of WS_2_ crystallization) the nanoscale structural evolution is again very similar with and without OctAm. The second step involves the formation of few layer intermediate structures with 3 nm stacking periodicity; a subsequent splitting is observed, leading to the final 3.4 nm multilayer, which can be related to the WS_2_ nanocrystals common to both mixtures, featuring wide angle XRD patterns in the top panels of Fig. S6a and b,† and a further multilayered structure which was again related to the organic moiety OlAm/OctAm. The latter disappeared with increasing annealing time, at the last stage of the reaction (the three topmost panels in Fig. S6c and d†).

Following the UV-vis spectra at the selected stages (Fig. 4f–j), from the WCl_6_ dissolution in OlAm (red curves) or in OlAm/OctAm mixture (black curves) to the reaction completion, we found that the characteristic optical spectra associated with 2D-WS_2_ NFLs were evident and anticipated at 215 °C for the OlAm/OctAm (Fig. 4h), whereas they were detectable only at 250 °C for the sole OlAm (Fig. 4i), in accordance with what was observed from XRD spectra. The shoulder found at 388 nm for both OlAm and OlAm/OctAm systems did not derive from any CS_2_–amine species or tungsten–amine derivative as observed by following the whole heating series recorded (Fig. S7a and b†). A reasonable interpretation is that such a peak was connected to the formation, at the early stages of the reaction course, of WS_2_ nano-nuclei with lateral dimensions below 2 nm (ref. 32, 33 and 41) as observed in similar systems^42–44^ and also confirmed by the optical feature of the crude aliquots (Fig. S7c and d†). We keep this optical feature as an indirect indication of WS_2_ small nuclei present in solution.

The evolution of the Fourier transform infrared (FTIR) spectra recorded from the same aliquots, corresponding to the flocculated products (see Experimental) for the two series, is reported in Fig. 4k–o. The complete FTIR evolution for both the procedures is reported in Fig. S12.† In a general view, the evolution of the FTIR spectra suggested the presence of oleylammonium chloride (based on the broad and intense –NH_3_^+^ stretching at 3100–2000 cm^−1^ and the intense peak at 1576 cm^−1^ ascribed to the symmetric bending/scissoring of –NH_3_^+^, Fig. S12a†) and oleylamine–thiourea (indicated by the broad but intense peak at 660–602 cm^−1^ ascribable to the symmetric stretching of C=S for the “free ligand” and interaction vibrations between –C=S and C–N– stretchings, respectively) from the early stages of the reaction.^45,46^ The signal ascribed to oleylamine–thiourea disappeared in flocculated samples at temperatures higher than 250 °C (Fig. 4m and n) when the reaction was performed in sole OlAm. Contrarily, in the OlAm/OctAm mixture, they were already absent at 215 °C (Fig. 4l–m), indicating that, in this latter case, the thermo-chemical decomposition of precursors was anticipated (as also supported by the thermogravimetric analysis (DSC-TGA) reported in Fig. S13†). Thus, the proceeding of the reaction caused a partial conversion of thiourea, while the signal of the long-chain amine persisted in the purified final product 2D-WS_2_ nanostructures, suggesting an oleylamine based residual organic capping. Overall, considering that in both the systems the NMR data did not evidence differences in the chemical pathway involved in the generation of colloidal NFLs, we could safely conclude that the growth of colloidal 2D-WS_2_-NFLs resulted anticipated in the case of OlAm/OctAm when compared to the only OlAm environment, likely because of the shorter chain length of OctAm, which eventually determined the larger lateral dimension of the as-obtained NFLs in analogy to what has been observed in other systems.^25,47,48^

## Conclusions

In summary, we have investigated in depth the dimensional control of solution processable 2D-WS_2_ nanocrystals in the mono-to-few layer thickness regime using an amine-based mild temperature non-hydrolytic solvothermal reaction. Two-dimensional NFLs were synthesized by the reaction of WCl_6_ in only OlAm and OlAm/OctAm with a solution of CS_2_ in OLAM, through the generation of oleyl–thiourea in solution, which gradually evolved into the relative thiolic tautomer during the reaction course. This chemical conversion with the associated 2D-WS_2_ nanocrystal evolution resulted to be amine content dependent and specifically it occurred under milder reaction conditions if a mixture of OlAm and OctAm was used. Moreover, the oleyl–thiourea formation requires *in situ* H_2_S generation that was responsible for the formation of initial WS_2_ tiny nanocrystals (nano-building blocks) embedded in an organic/inorganic self-assembled lamellar precursor. Thus, we believe that the bi-dimensional assembly of nano-nuclei, generated within the soft-template precursor, subsequently evolved into extended 2D NFLs. The stability/reactivity of WS_2_ nanoclusters depends on the chemical nature (which is reflected in the precursor reactivity) and steric hindrance of the alkylamine composition adopted for the synthesis, which strongly influences the dimension of the nanocrystals finally achieved. Further investigations are needed to rationalize the chemistry that dominates the resulting different morphologies as a consequence of a different amine composition in the reaction media. We believe that our findings can foster the development of a more reliable and effective synthesis of 2D-TMD materials.

## Methods

### Materials

Tungsten(vi) chloride (WCl_6_, 99.9%), 1-octadecene (C_18_H_36_ or ODE, 90%), oleyl amine (C_17_H_35_NH_2_ or OlAm, 70%) and octyl amine (CH_3_(CH_2_)_6_CH_2_NH_2_ or OctAm, 99%) were purchased from Sigma-Aldrich. All solvents used were anhydrous and of analytical grade. Chloroform, 2-propanol, and carbon disulfide (CS_2_) were purchased from Sigma-Aldrich. Dried acetone (max. 0.0075% H_2_O) was purchased from SeccoSolv. Dried and deuterated toluene (Tol-d8) with 0.03% (v/v) TMS was purchased from Sigma-Aldrich. All chemicals and solvents were used as received without any further purification. OlAm and ODE were individually degassed at 80 °C for 1 h, and then repeatedly purged with nitrogen and stored in a N_2_-protected glovebox prior to use.

### Synthetic protocol for 2D-WS_2_ NFLs

All syntheses were carried out under air-free conditions using a standard Schlenk line setup.

In a typical preparation of 2D-WS_2_ nanoflakes with a lateral dimension of <5 nm, 100 mg (0.25 mmol) of WCl_6_, 1 mL (3.1 mmol) of degassed ODE and 1 mL (3 mmol) of degassed OlAm were loaded into a three-neck flask under a N_2_ atmosphere in a glovebox, and then allowed to mix under vigorous stirring at room temperature until the initially dark suspension rapidly converted into an optically clear yellow/reddish solution. The system was degassed (20 mTor) for 60 min at 130 °C under vigorous stirring until a solution of 60 μL (1 mmol) of CS_2_ in 2.5 mL (∼7.6 mmol) of degassed OlAm was added to the mixture. The resultant mixture was aged at 130 °C for 1 h, and then slowly heated to ∼250 °C under N_2_ flow at a ramp rate of ∼5 °C min^−1^ and kept at this temperature for 2 h.

To grow 2D-WS_2_ nanoflakes with a lateral dimension of around ∼25–30 nm, 1 mL (6 mmol) of OctAm was added into the mixture described above, without any other variation in the procedure.

### Extraction and purification procedures

After the synthesis, the reaction mixture was cooled down to ∼40–50 °C and swiftly transferred to a N_2_-protected glovebox. At this point, 15 mL of dried 2-propanol/acetone mixture (2 : 1 v/v) was added to induce flocculation of the nanocrystalline product. The resulting suspension was centrifuged at 3000 rpm for 10 min, and then the collected precipitate was separated from the supernatant and purified through two cycles of dissolution in chloroform (or toluene) and re-precipitation with only acetone. Finally, the as-purified product was entirely re-dispersed in ∼5–10 mL of desired nonpolar solvent (toluene or chloroform) to yield optically clear colloidal dark brown or black solutions that were colloidally stable for months, if stored in an air-protected environment.

### Powder X-ray diffraction (XRD)

Measurements were performed with a D8-Discover Bruker diffractometer (2.2 kW) equipped with a CuKα (*λ* = 1.54 Å) source (operated at 40/40 mA/kV), a Goebel mirror, an Eulerian cradle goniometer, and a scintillator detector. XRD patterns were collected at a fixed incident angle of 2° while moving the detector over a 2*θ* range of 1–120° with a step size of 0.05°. A LaB_6_ NIST standard was used to measure the instrumental resolution function. Samples were prepared by spreading concentrated toluene solutions of the as-purified colloidal NPLs or NFLs on a silicon substrate in a glovebox and allowing the solvent to evaporate. The substrate was transferred to the diffractometer and the samples were measured under an ambient atmosphere at room temperature. The XRD profiles were simulated with the Debye equation,^29,30,49^ by summing all over interatomic distances of WS_2_ nanoparticles in the 1T′^8,50^ and 2H^28^ crystal phases with variable number of unit cells along the *a*-axis [100] and the *c*-axis [001]. The obtained simulated XRD profiles derived from the combination of 1T′ and 2H nanocrystals, with suitable weight fractions and crystalline domain sizes in terms of the number of unit cells along the main crystallographic axes (*a*, *b*, and *c*); specifically 83.3% of 1T′ 1 × 10 × 10 + 0.1% of 1T′ 1 × 20 × 20 + 0.8% of 2H 20 × 20 × 1 + 2.8% of 1T′ 2 × 10 × 10 + 13% of 1H 40 × 40 × 1. By reducing particle thickness along a specific crystallographic direction, the resulted simulated XRD patterns derived from the almost complete suppression of the main reflections, 100 or 002 peaks at about 1 Å^−1^, characteristic of the 1T′ or 2H phases of the bulk material, respectively. expected to be predominant for the bulk structure.

### Transmission electron microscopy (TEM) and high angle annular dark-field scanning transmission electron microscopy (HAADF STEM)

Conventional TEM analyses were performed with a JEOL JEM 1400Plus microscope operating at 120 kV, with a LaB_6_ source and a GATAN Orius SC600 CCD camera. High resolution HAADF STEM images were acquired by using the FEI Titan 50–300 PICO microscope installed at the Ernst Ruska-Centre for Microscopy and Spectroscopy with Electrons (ER-C), Jülich (Germany), equipped with a Schottky type high-brightness electron gun (FEI X-FEG), here operated at 80 kV, a monochromator unit, a Cs probe corrector (CEOS DCOR), a Cs-Cc achro-aplanat image corrector (CEOS CCOR+), and a post-column energy filter system (Gatan Quantum 966 ERS) as well as a 16 megapixel CCD system (Gatan UltraScan 4000 UHS)

### UV-vis measurements

UV-vis absorptions of 2D-WS_2_ nanoparticles dispersed in CHCl_3_ were recorded using a Varian Cary300 spectrophotometer. For the aid, bare aliquots collected from the reaction and diluted in dried CHCl_3_ and the relative clean/precipitated aliquots were measured for comparison. The aliquots were precipitated by following the same procedure adopted for the final nanocrystal product.

### Fourier transform infrared spectroscopy (FTIR)

FT-IR spectra were recorded over the 4000–400 cm^−1^ wavenumber range at a resolution of 4 cm^−1^ using a Bruker Equinox 70 spectrometer. Samples were prepared by drop-casting a concentrated nanocrystal solution onto a dedicated Si-substrate in an air protected glove box, and then allowing the solvent to rapidly evaporate in the air protected glovebox. The sole Si-substrate was used for instrumental baseline correction purposes.

## Nuclear magnetic resonance (NMR)

Experimental samples were prepared by diluting 100 μL of reaction mixture in 600 μL of deuterated toluene-d_8_. The oleylamine hydrochloride salt (OlAm·HCl) was produced by mixing a 5% solution of OLAM in chloroform with 5% HCl aqueous solution. The hydrochloride salt thus formed is almost insoluble in water but soluble in the organic phase. The removal of excess solvent produced a viscous oil, which was used without further purification. The removal of solvent produced a viscous oil, which was used without further purification.^51^ NMR measurements were performed on a spectrometer operating at 600 and 150 MHz for ^1^H and ^13^C, respectively. The temperature was controlled at 25 ± 0.1 °C. The 2D NMR spectra were obtained by using standard sequences. The spectral width used was the minimum required in both dimensions. Proton gCOSY (gradient COrrelated SpectroscopY) 2D spectra were recorded in the absolute mode acquiring 8–16 scans with a 1 s relaxation delay between acquisitions and 4k data points for each of the 200 FIDs. 2D TOCSY (TOtal Correlation SpectroscopY) spectra were recorded acquiring 8–32 scans with a 1 s relaxation delay, 200 increments, 4k data points and a mixing time of 120 ms. The ROESY (Rotating-frame Overhauser Enhancement SpectroscopY) spectra were recorded in the phase-sensitive mode, by employing a mixing time of 0.2 s; the relaxation delay was maintained at 1 s; 200 increments of 16 scans and 4k data points each were collected. The gradient gHSQC (gradient Heteronuclear Single Quantum Coherence) spectra were obtained in 64 scans for each of the 200 increments.

## Conflicts of interest

There are no conflicts of interest to declare.

## Supplementary Material

NA-001-C9NA00279K-s001
