# Supplementary material for: Mechanistic insight into the formation of colloidal WS2 nanoflakes in hot alkylamine media
Source: Nanoscale Adv. 2019 May 24;1(7):2772–82. doi: 10.1039/c9na00279k (PMC9418938; doi:10.1039/c9na00279k)
Supplement: NA-001-C9NA00279K-s001 [file NA-001-C9NA00279K-s001.pdf]

Electronic Supplementary Material (ESI) for Nanoscale Advances.
This journal is © The Royal Society of Chemistry 2019

Supporting info

Mechanistic Insight into the Formation of Colloidal WS_2 Nanoflakes in Hot Alkylamine Media.

Riccardo Scarfiello,[1] Andrea Cesari,[2] Davide Altamura,[3] Sofia Masi,[1,6] Concetta Nobile,[1] Federica Balzano,[2] Cinzia Giannini,[3] Vincenzo Grillo,[4] Amir H. Tavabi,[5] Rafal E. Dunin-Borkowski,[5] Gloria Uccello-Barretta,[2] P. Davide Cozzoli[1,6] and Aurora Rizzo[1]

[1] CNR NANOTEC, Institute of Nanotechnology, c/o Campus Ecotecne, via Monteroni, 73100 Lecce, Italy;

[2] Department of Chemistry and Industrial Chemistry, University of Pisa, via Moruzzi 13, 56124 Pisa, Italy;

[3] IC CNR, Institute of Crystallography, via Amendola 122/O, I-70126 Bari, Italy;

[4] Centro S3, CNR Istituto Nanoscienze, via Campi 213/A, 41125 Modena, Italy;

[5] Ernst Ruska-Centre for Microscopy and Spectroscopy with Electrons, Forschungszentrum Jülich, Germany;

[6] Department of Mathematics and Physics E. De Giorgi, University of Salento, via per Arnesano, 73100, Lecce, Italy.

Table of Contents

I. *Complementary XRD simulations* *S2*

II. *Complementary X-Ray spectra* *S5*

III. *Complementary UV-Vis extinction spectra* *S7*

IV. *Complementary NMR spectra* *S8*

V. *Complementary FTIR spectra* *S10*

VI. *Thermogravimetric (TGA) and Differential Scanning Calorimetric (DSC) analysis* *S12*

I. <u>*Complementary XRD simulations:*</u>

Simulations of the X-ray diffraction pattern, based on the Debye equation, show how the typical saw-tooth profile is only recovered when the number of unit cells in the crystalline domain is reduced along one axis: the *a* axis in the case of the 1T' phase and the *c* axis in the case of the 2H phase.

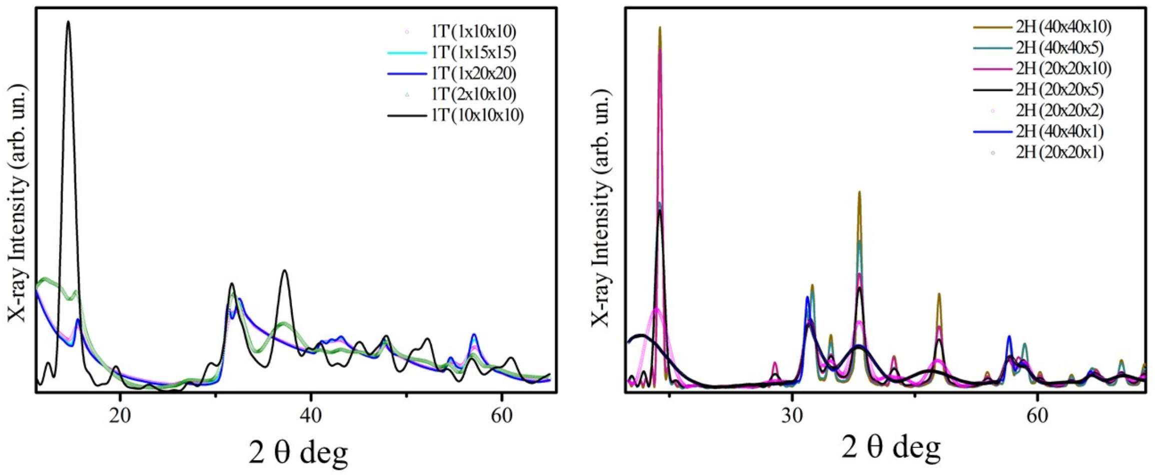

Figure S 1 Simulated XRD patterns calculated for crystalline domains with different dimensions along the *a* axis and along the *c* axis for the 1T' (pattern on the left) and 2H (pattern on the right) crystal phases. The number of unit cells making the crystals along the *a*, *b* and *c* axes, respectively, are reported in the legend.

Therefore, in order to damp the first (highest) peak and broaden the other ones, so as to match the experimental profile, simulations with no more than 2 unit cells along the *a* axis (in the case of the 1T' phase) or the *c* axis (in the case of the 2H phase) are considered in the following.

Once suppressed the first peak, the next representative range to be considered is between 30° and 60° (2θ). It is then readily recognized, based on simulations comparing the 1T' and 2H phases in Fig. S2, that the peaks around 42.5° and 47.5° (2θ) are characteristic of the 1T' phase, whose presence is therefore established. Moreover, such peaks are quite weak and broad, resulting from the convolution of several reflections, while another characteristic peak of the 1T' phase at 54.6° is even missing; so that an even smaller domain in the (*b*,*c*) plane can be expected.

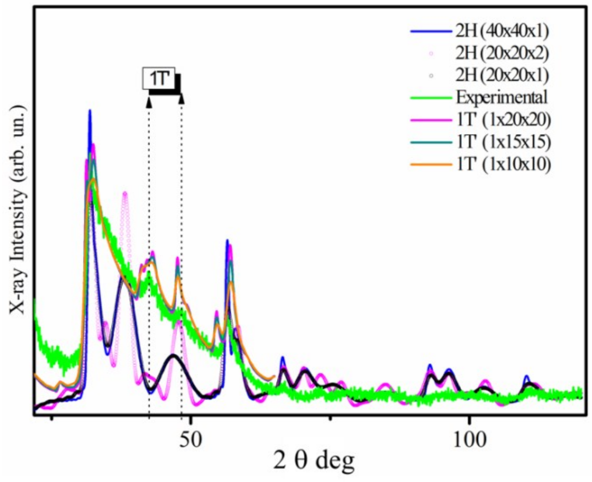

Figure S2 Simulated XRD patterns calculated for crystalline domains with different dimensions along the *a* and *c* axes for the 1T' and 2H crystal phases, showing how the two marked peaks at 42,5° and 47,5° are mainly related to the 1T' crystal phase.

On the other hand, a close inspection (Fig. S3) of the regions around the two main peaks at 32° and 57°(2θ), reveals a much better match between calculation and experiment for the 2H phase (Fig. S3c,d). Moreover, in the zoomed regions around the two peaks, it is clearly seen that a number of 1T' unit cells larger than 10 along the *b* and *c* axes would lead to a splitting of the first peak (Fig. S3a); whereas a number of 2H unit cells larger than 20 along the *b* and *c* axes would lead to much sharper and intense peaks (Fig. S3c,d).

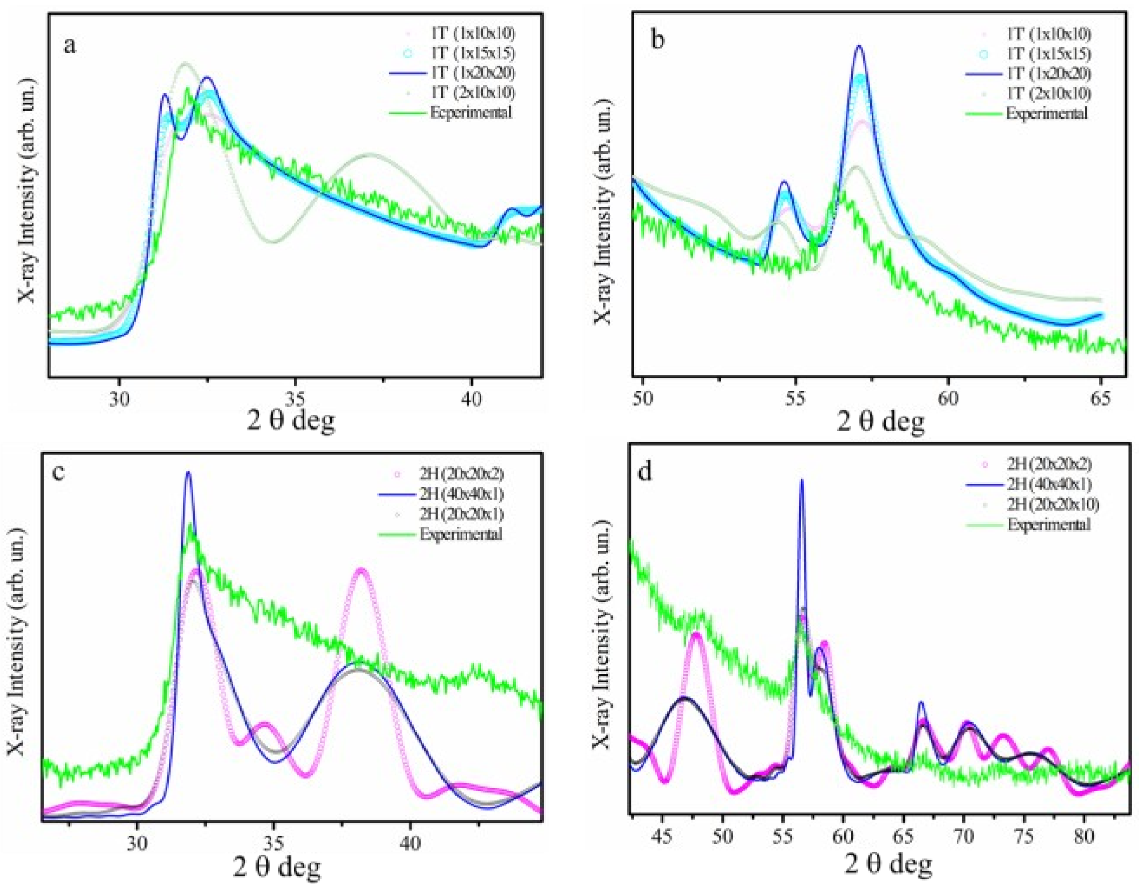

Figure S3 Simutaled XRD patterns showing how peak profiles in the different angular regions are affected by crystalline domain size of 1T' and 2H phases.

As a natural consequence, the experimental X-ray scattering profile can be expected to result from a superposition of the profiles relevant to 1T’ and 2H two-dimensional nanocrystals with suitable weights and crystalline domain size (Fig. S4). However, no combinations of two solutions (for any size and weight fraction of the two phases) are sufficient to reproduce the experimental profile, meaning that polydispersion has to be taken into account, with smaller domains mainly contributing to the diffuse scattering in the range 32°-40°, and larger domains leading to a sharpening of the two main peaks (Fig S4). Combinations of three or more profiles are then considered; the results for the best overall agreement (Fig. S5) suggest that the main scattering contributions come from 1T’ NCs with 1x10x10 unit cells and 2H NCs with 40x40x1 unit cells, being the weight of any additional 1T’ contribution required to be negligible.

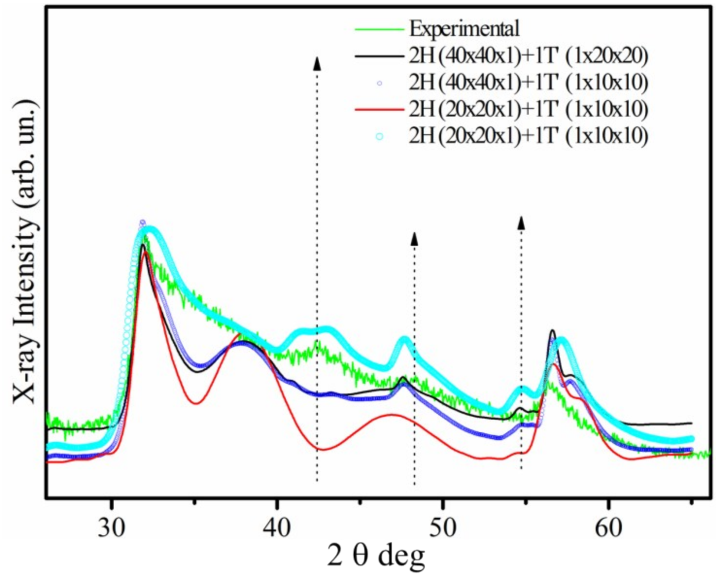

Figure S4 Simulated XRD profiles for different combinations of 1T’ and 2H two-dimensional nanocrystals, with suitable weights and crystalline domain size.

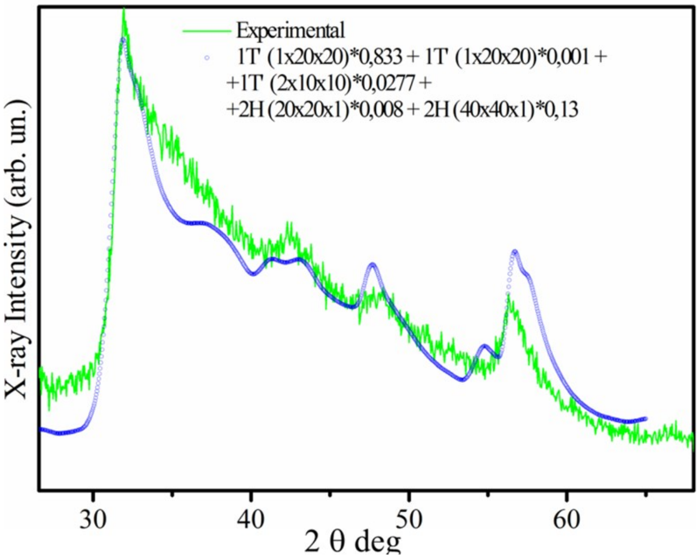

Figure S5 Simulated XRD profiles resulting from the combination of 1T’ and 2H two dimensional nanocrystals, leading to the best agreement with experiment. The relative abundance of each nanocrystal considered in the population is reported in the legend as a multiplication factor for each term contributing to the calculated scattering profile.

II. ***Complementary X-Ray spectra:***

To support the structural evolution described by the wide angle XRD patterns reported in Fig. 4a-e, we present wide and small angle XRD patterns in Fig. S6a,b, and Fig. S6c,d respectively, in order to describe a more detailed structural evolution of recorded intermediate aliquots. Aliquots recorded at 130 °C and 160 °C for both OlAm and OlAm/OctAm mixture show featureless XRD patterns, ascribed to amorphous material or composed of too small crystalline domains. Considering the aliquots recorded at the 215 °C, the scenario completely changes because when OlAm/OctAm mixture is used (black plot), a XRD pattern clearly ascribable to the WS_2 nanosheets formation is detected, whereas an amorphous-like pattern is still characterizing the sample generated in sole OlAm (red plot) that eventually evolves in a pattern characteristic of heterophased WS_2 nanomaterials only heating up to 250 °C. Small-angle XRD patterns related to isolated metalorganic intermediate generally indexed as $WCl_{(6-x)}$:ODE:$OlAm_x$:CS_2 and $WCl_{(6-x-y)}$:ODE:$OlAm_x$:$OctAm_y$:CS_2 (Fig. S6c,d) give precious information on time/temperature evolution of the lamellar layered mesostructures. The d-spacing (periodicity) experimentally observed, reported on top right of every individual frame, prior to the CS_2 injection, is 4.25 nm for $WCl_{(6-x)}$:ODE:$OlAm_x$ (Fig. S6c, bottom spectra) and is reduced to 3.92 nm for $WCl_{(6-x-y)}$:ODE:$OlAm_x$:$OctAm_y$ (bottom spectra in Fig. S6d), thus compatible with a two or more inorganic layers spaced apart by a double organic layer arranged with a tilting angle around 50° and 33° respectively, considering the interlayer distance and carbon chain length[1, 2] which compose the hybrid organic/inorganic lamellar phase. Upon injection of CS_2 precursor and heating at 130 °C, with the simultaneous formation of oleyl-thiourea and the in situ releasing of H_2S, a characteristic interlayer distance d of 3.4 nm is detected for both systems, which can be approximately estimated as the sum of the ~0.7 nm thick newly formed WS_2 nanoclusters, the two extra C-N (0.133 nm)[3] and N-S (0.171 nm)[3] bonds per OlAm molecule introduced in the structure as consequence of thiourea formation, and a double layer of OlAm constituent of formed thiourea (~2 nm)[4] with a tilt angle of ~30°. The same periodicity of 3.4 nm has been found in the final products, where larger particles (in the stacking direction) can be expected, based on the sharpening of corresponding peaks in the wide angle range. The recovered periodicity of 3.4 nm is ascribable to a super-structure of surface capped planar nanocrystals arranged in turbostratic organization (Fig. S6c,d, top spectra).

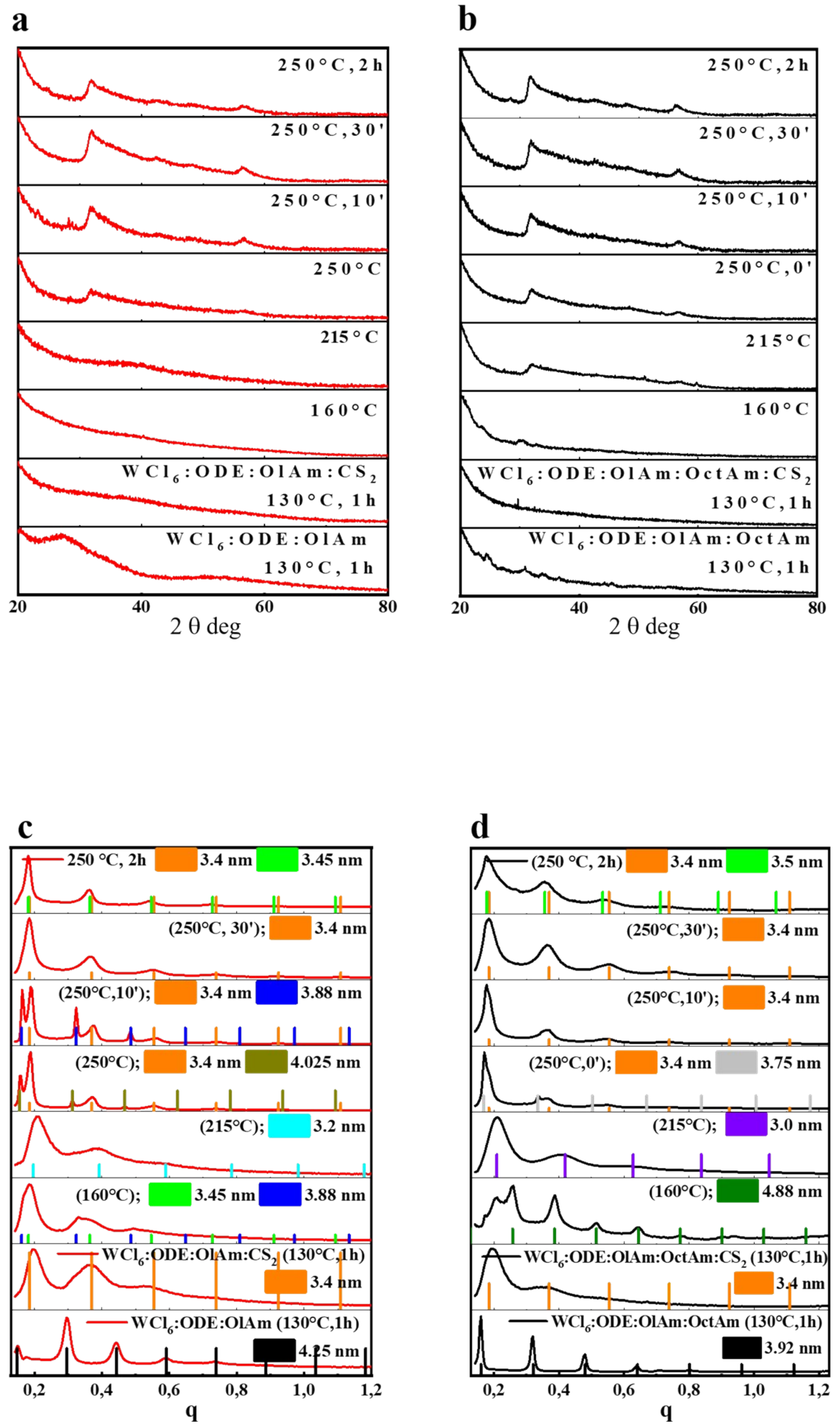

Figure S6 All measurements are performed on purified sample by following the experimental procedure, then dissolved in anhydrous $CHCl_3$ and casted on Si-substrate in N_2 atmosphere. (a) Wide Angle (20°<2θ<80°) XRD (crystal structure) evolution recorded on time/temperature scheduled aliquots derived from synthesis of 2D-WS_2 nanoplatelets generated in only OlAm environment, withdrawn at the specific reaction condition reported in the relative legend; (b) Wide Angle (20°<2θ<80°) XRD (crystal structure) evolution recorded on time/temperature scheduled aliquots derived from synthesis of 2D-WS_2 nanoplatelets generated in OlAm/OctAm (1:2 mmol ratio) environment, withdrawn at the specific reaction condition reported in the relative legend; (c) Small Angle ($0.1<q = (4\pi/\lambda)\sin\theta < 1.2$ Å^{-1}) XRD (nanoscale structure) evolution reporting inter-planar distances on x-axis, recorded on time/temperature scheduled aliquots derived from synthesis of 2D-WS_2 nanoplatelets generated in only OlAm environment, withdrawn at the specific reaction condition reported in the relative legend; (d) Small Angle ($0.1<q = (4\pi/\lambda)\sin\theta < 1.2$ Å^{-1}) XRD (nanoscale structure) evolution reporting inter-planar distances on x-axis, recorded on time/temperature scheduled aliquots derived from synthesis of 2D-WS_2, nanoplatelets generated in OlAm/OctAm (1:2 mmol ratio) environment, withdrawn at the specific reaction condition reported in the relative legend. In (c) and (d), the periodicities correlated to the small angle equally spaced diffraction peaks are reported in the legends of the individual plots with relevant colour code. In caption are reported the experimental condition where each sample has been exposed (a-d) and the corresponding periodicities of equally spaced (in reciprocal space units) peaks (c,d).

III. Complementary UV-Vis extinction spectra:

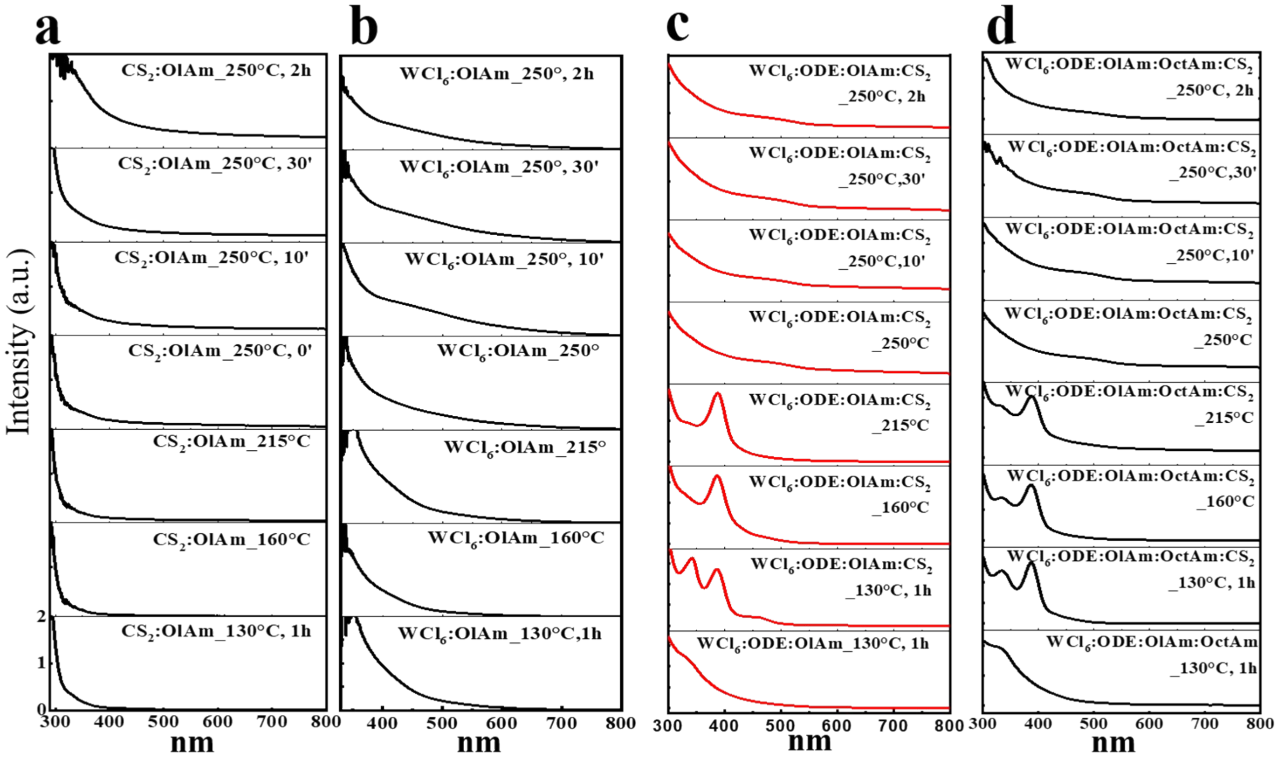

Figure S7 UV-Vis extinction measurements on time/temperature scheduled aliquots withdrawn at the specific reaction condition reported in the relative legend and derived from (a) CS_2/OlAm (0.5:3 mmol) solution (b) WCl_6/OlAm (0.25:3 mmol) (c) WCl_6/ODE/OlAm/CS_2 (0,25:3:3:1) and (d) WCl_6/ODE/OlAm/OctAm/CS_2 (0,25:3:3:6:1), obtained from aliquots non precipitated, dispersed in anhydrous $CHCl_3$ in air protected atmosphere.

IV. Complementary NMR spectra:

In order to prove that the chemical path reported in scheme 1 can be extended also for nanocrystal synthetized in OlAm/OctAm, we have reported here the reaction products involved with an experimental reference model formulated by using two equivalents of OctAm or one equivalent of OctAm and one of OlAm with one equivalent of CS_2. No significant differences were detected in the positions and shape of the signals of α-CH_2N protons of OctAm or OlAm/OctAm derivatives, which is concentration-affected as dictated by slight shifts in peak position (Fig. S8).

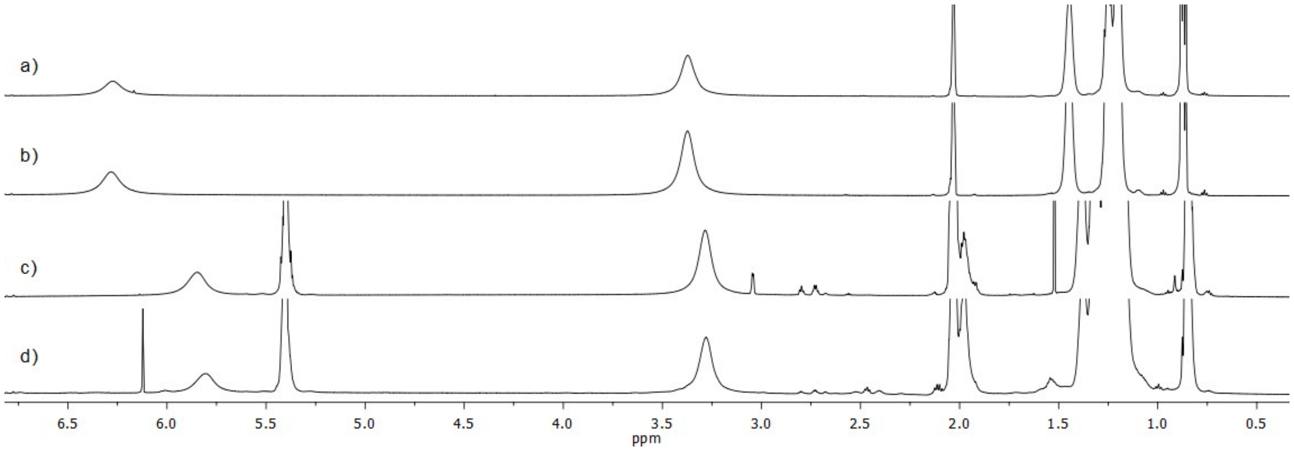

Figure S8 ^{1}H NMR spectrum (600 MHz, toluene-d_8, 25 °C) of: a) CS_2/OctAm (1:2) aged at 130 °C for 1h b) CS_2/OctAm (1:2) aged at 250 °C for 1 h c) CS_2/OlAm/OctAm (1:1:1) aged at 130 °C for 1 h d) CS_2/OlAm/OctAm (1:1:1) aged at 250 °C for 1 h.

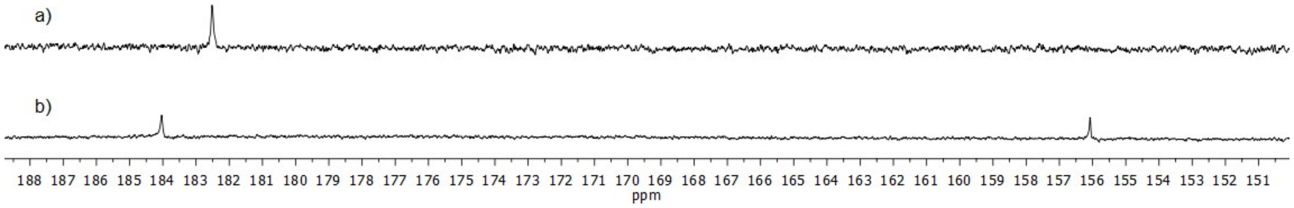

Figure S9 Portion of ^{13}C NMR spectrum (150 MHz, toluene-d_8, 25 °C) of: a) CS_2/OlAm (1:2) aged at 250 °C, b) WCl_6/CS_2/OlAm (0.25:4:8.5) aged at 250 °C.

In the mixture WCl_6/CS_2/OlAm (0.25:4:8.5) aged at 130 °C, the formation of oleylammonium species, referred as compound **2** (Scheme 1 in main text), was clearly observed, originating the low frequency shifted CH_2-N centered at 3.04 ppm and a very broad high frequency shifted signal at 8.70 ppm (Fig. S10b). The formation of compound **2** was ascertained on the basis of the integration ratio between the two above said signals (2 to 3) and the absence of any new quaternary carbon in the ^{13}C NMR spectrum. The spectral pattern of such a species reproduced the one of the oleylamine hydrochloride salt (OlAm•HCl, see Fig. S10a), suggesting that WCl_6 is responsible for the formation of HCl. Ammonium salt is influenced by the presence of the metal,[(5)] since the relaxation times of ammonium species in nanoparticles systems are lower (T_1 = 0.31 s for CH_2-N) if compared to pure OlAm•HCl salt (0.55 s). It is noteworthy that the amount of ammonium salt

formed in the final nanoparticle product is correlated to the amount of WCl_6, as demonstrated by comparing ^1H NMR spectra of mixtures at differrent WCl_6 to amine ratios (Fig. S10b-c).

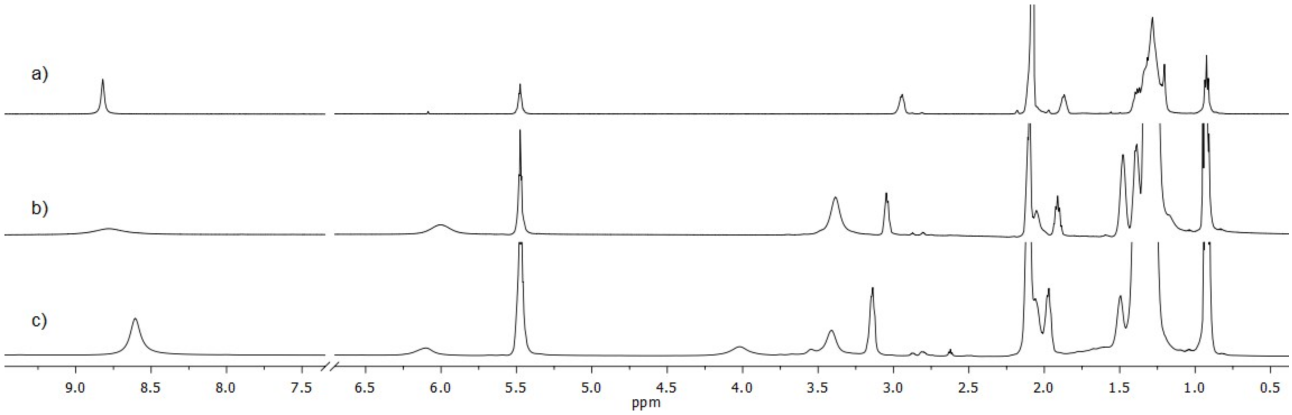

Figure S10 ^1H NMR spectrum (600 MHz, toluene-d_8, 25 °C) of: a) oleylamine hydrochloride salt (OlAm·HCl), b) WCl_6/CS_2/OlAm (0.25:4:8.5) aged at 130 °C for 1 h, c) WCl_6/CS_2/OlAm (0.67:4:8.5) aged at 130 °C for 1 h.

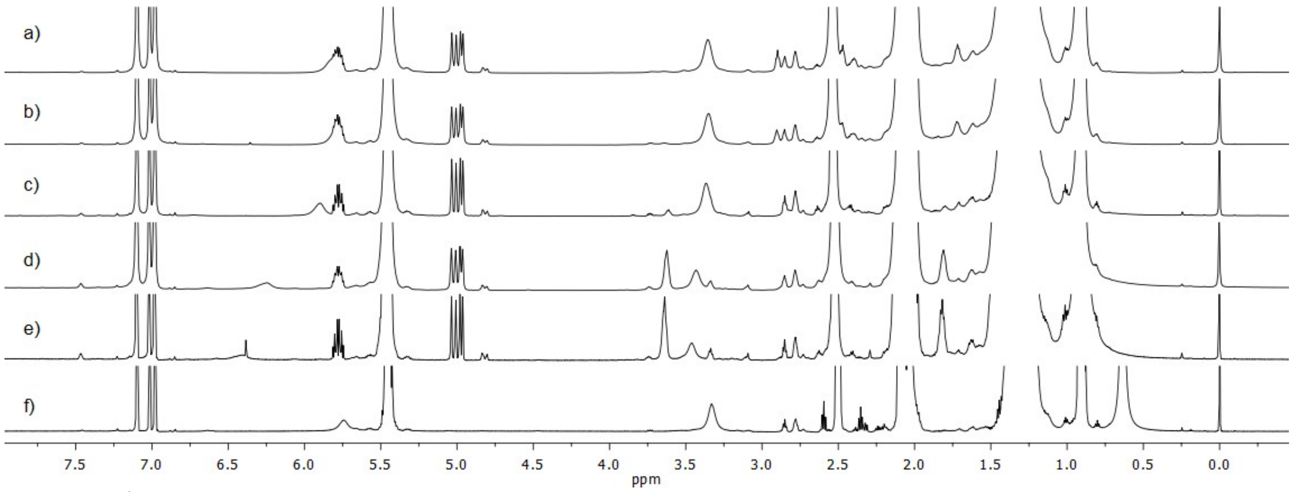

Figure S11 ^1H NMR spectrum (600 MHz, toluene-d_8, 25 °C) of: WCl_6/ODE/OLAM/CS_2 in 3-5 nm WS_2 NFLs synthetic condition (0.25:3:3:1) a) (130 °C, 1h), b) (250 °C, 0 min), c) (250 °C, 10 min), d) (250 °C, 1h), e) (250 °C, 2h) and f) OlAm/CS_2 in synthetic condition (3:1, 250 °C, 2h) .

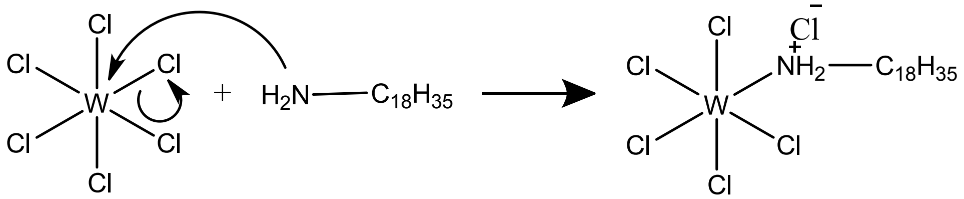

Scheme S1 Chemical pathway proposed involved for the formation of oleyl ammonium chlorine salt coordinated to tungsten precursor, as a consequence of dissolution of WCl_6 with OlAm

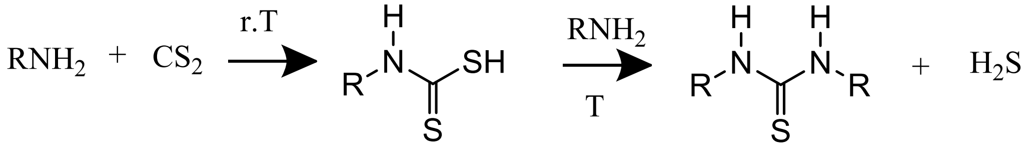

Scheme S2 Chemical pathway proposed involved between CS_2 and OlAm.

V. Complementary FTIR spectra:

The FTIR characterization confirms that when the reaction is performed in sole OlAm, the broad but intense peak at 660-602 cm^{-1} ascribable to symmetric stretching of C=S for "free ligand" and interaction vibrations between -C=S and C-N- stretchings respectively, disappears at temperature higher than 215 °C (Fig. 3m – Fig. 3n). Contrarily, in OlAm/OctAm mixture, they are absent already at 215 °C (Fig. 3l – Fig. 3m), indicating that in this latter case, the thermo-chemical decomposition of precursors is anticipated.

When anhydrous OlAm and WCl_6 were heated together (130 °C, 1h; Fig. S12a) an appreciable degree of protonated amine in the reaction mixture, arising from the broad and intense $-NH_3^+$ stretching at 3100 – 2000 cm^{-1}, which overlapped with the fine structure covering all the symmetric and asymmetric C-H stretchings of hydrocarbons chains, becomes evident. The intense peak at 1576 cm^{-1} can be ascribed with the symmetric bending/scissoring of $–NH_3^+$. Peaks at 1465-1431 cm^{-1} are ascribed as $-CH_3$ and $-CH_2$ scissoring and the signal at 1066 cm^{-1} can be ascribed as -C-N stretching signal. The scenario does not significantly differ when a combination of OlAm/OctAm (1:2 molar ratio) is adopted to dissolve by complexation the halogenated tungsten precursor (Fig. S12i). It is worth to mention that vibration frequencies involving amino group appear at lower wavenumber due to the combined effect of the interaction with the lamellar intermediate and the possibility to have intermolecular interaction for the solid phase sample. The frequencies ascribed to the hydrocarbon chain (σ(C-H) 2954-2853 cm^{-1}) appear at the same values in both samples and in the neat amines. When CS_2-OlAm solution is injected in the flask, the mixture is stirred for 1h before gradually reaches the target temperature of 250 °C. In the related FTIR spectra (Fig. S12b-d and Fig. S12j-k) the σ_s(N-H) at 3236 cm^{-1} and σ_{as}(N-H) 3199 cm^{-1} (symmetric and asymmetric stretchings) of excess of unreacted primary amine are visible and could reasonably contain also stretchings related to the secondary amine contained in oleylamino-thiourea. Concerning with NMR spectroscopy (Fig. S10), there is an unneglectable portion of protonated amine. The sharp and intense signal at 1576 cm^{-1} ascribed with the symmetric bending/scissoring of $–NH_3^+$ disappear when the reaction temperature rises over 215 °C or 160 °C for only OlAm and OlAm/OctAm respectively; the δ(C=C) at 1640 cm^{-1} becomes predominant and remains unaltered until the end of the reaction. The sharp and intense signal at 1465 cm^{-1} is ascribed to δ_s CH_2/CH_3 (scissoring), together with the C-C vibrations at 722 cm^{-1}, can be recognized for the whole FTIR evolution.

The developing or thermodynamic stabilization of the thiolic tautomer concomitant with the developing of the nanocrystalline surfaces suggests the formation of $S \rightarrow W_{NCs}$ bond which increases the contribution of the double bond character for the nitrogen-to-carbon bond and a greater carbon-to-sulfur single bond character (Fig. S12q)[6]. A new couple of peaks at 965 cm^{-1} and 909 cm^{-1} increase in intensity with the proceeding of the reaction time and it has been associated to the stretching mode of new N-C-N contained in the thiolic species stabilized by the presence of WS_2 nanostructures through the coordinating on the W metal. Those signals show different relative intensity in sample synthesized in sole OlAm (Fig. S12o, red plot) and in OlAm/OctAm 1:2 molar ratio mixture (Fig. S12o, black plot).

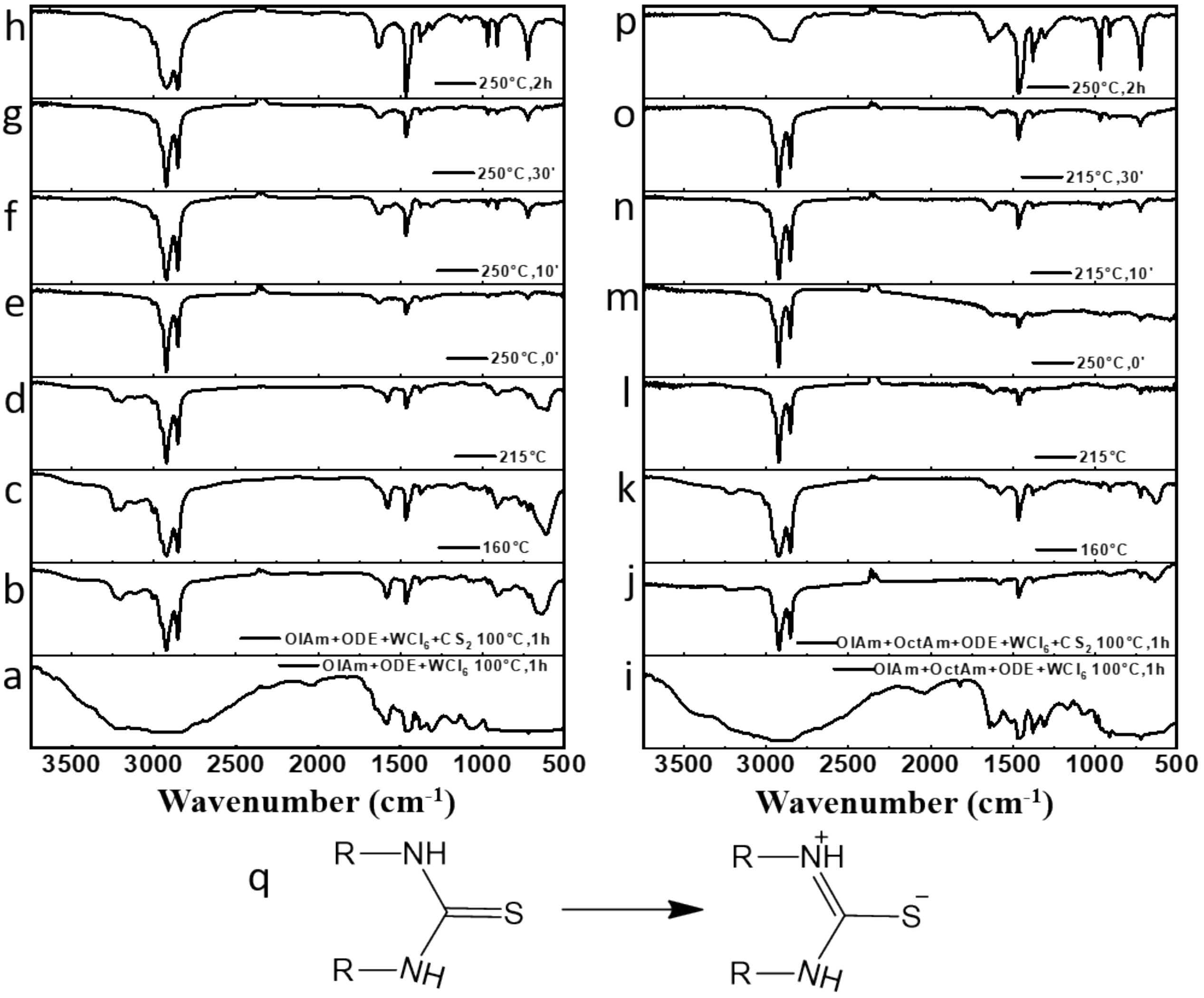

Figure S12 Full range FTIR measurements on time/temperature scheduled aliquots withdrawn at the specific reaction condition reported in the relative legend from (a) WCl_6/ODE/OlAm (0,25:3:3), (b-h) WCl_6/ODE/OlAm/CS_2 (0,25:3:3:1), (i) WCl_6/ODE/OlAm/OctAm (0,25:3:3:6), (j-p) WCl_6/ODE/OlAm/OctAm/CS_2 (0,25:3:3:6:1), casted in air protected glove-box after purified solution. (q) Scheck of two possible resonance structures of generic $(R\text{-}NH)_2$-thiol.

VI. Thermogravimetric (TGA) and Differential Scanning Calorimetric (DSC) analysis:

TGA thermograms for both synthetic systems, sole-OlAm and OlAm/OctAm (1:2 molar ratio), are reported in Fig. S13. Mass loss begins around 200 °C and concludes at 325-350 °C. The mass losses correlate pretty well with those expected for the generation of WS_2 NCs starting from corresponding original mixture, which are composed by ODE, OlAm and OlAm-CS_2 derivate, for more than 95% of their weight. In the same figure are reported the corresponding DSC thermograms for the same sole-OlAm and OlAm/OctAm (1:2) synthetic mixtures. Here, there are multiple peaks but we focus on the exothermic heat flow at 250 °C for NFLs (OlAm/OctAm 1:2 mixture) and 275 °C for NFLs (sole OlAm mixture), which are comparable with the experimental temperatures adopted during the NCs synthesis. The nucleation events in the presence of same volume of OlAm/OctAm appeared to be slightly anticipated compared to the sole-OlAm system, suggesting a key effect of octylamine of the reaction kinetics.

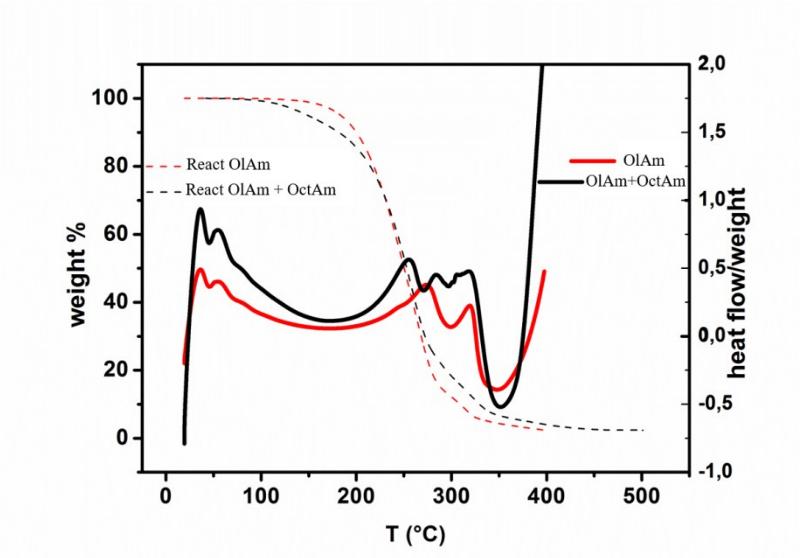

Figure S13 TGA (solid line) and DSC (dashed lines) of reaction mixture WCl_6/ODE/OlAm/CS_2 (0,25:3:3:1) (red plot), and WCl_6/ODE/OlAm/OctAm/CS_2 (0,25:3:3:6:1) (black plot).

1. Chen D, Sugahara Y. Tungstate-Based Inorganic−Organic Hybrid Nanobelts/Nanotubes with Lamellar Mesostructures: Synthesis, Characterization, and Formation Mechanism. Chemistry of Materials. 2007;19(7):1808-15.
2. Lagaly G. Interaction of alkylamines with different types of layered compounds. Solid State Ionics. 1986;22(1):43-51.
3. Kunchur NR, Truter MR. 517. A detailed refinement of the crystal and molecular structure of thiourea. Journal of the Chemical Society (Resumed). 1958(0):2551-7.
4. Borges J, Ribeiro JA, Pereira EM, Carreira CA, Pereira CM, Silva F. Preparation and characterization of DNA films using oleylamine modified Au surfaces. Journal of Colloid and Interface Science. 2011;358(2):626-34.
5. Brisdon BJ, Fowles GWA, Osborne BP. The reaction of tungsten(VI) chloride with some aliphatic amines. Journal of the Chemical Society (Resumed). 1962(0):1330-4.
6. Yamaguchi A, Penland RB, Mizushima S, Lane TJ, Curran C, Quagliano JV. Infrared Absorption Spectra of Inorganic Coördination Complexes. XIV. Infrared Studies of Some Metal Thiourea Complexes1a. J Am Chem Soc. 1958;80(3):527-9.
